# Complete chloroplast genome sequence of *Dysosma versipellis* (Berberidaceae), a rare and threatened species endemic to China

**DOI:** 10.1080/23802359.2019.1693928

**Published:** 2019-11-22

**Authors:** Yancai Shi, Haiping Yuan, Rong Zou, Bingbing Liu

**Affiliations:** a Institute of Loess Plateau, Shanxi University, Taiyuan, Shanxi, China;; b Guangxi Institute of Botany, Guangxi Zhuang Autonomous Region and Chinese Academy of Sciences, Guilin, China;; c Shanxi pharmaceutical vocational college, Taiyuan, Shanxi, China

**Keywords:** Dysosma, chloroplast genome, phylogenetic analysis

## Abstract

*Dysosma versipellis* (Berberidaceae) is a rare and threatened medicinal herb endemic to subtropical China. Here, we first report and characterize its complete chloroplast genome based on Illumina paired-end sequencing data. The complete plastid genome was 156,735 bp, which contained inverted repeats (IR) of 25,925 bp separated by a large single copy (LSC) and a small single copy (SSC) of 86,514 bp and 18,371 bp, respectively. The cpDNA contains 133 genes, comprising 86 protein-coding genes, 37 tRNA genes, 8 rRNA genes, and 2 processed pseudogenes. The overall GC content of the plastome is 38.5%. The phylogenetic analysis of 17 selected chloroplast genomes demonstrated that *D. versipellis* is closely related to the congeneric *D. pleiantha*.

*Dysosma versipellis* (Hance) M. Cheng ex Ying, which belongs to the subfamily Podophylloideae Eaton within the Berberidaceae family of Ranunculales, is a perennial herb and mainly distributed in temperate deciduous forests region endemic to subtropical China (Zhang et al. 1991). It is a well-known traditional Chinese medicine and has been used as a general remedy for the treatment of snakebites, weakness, condyloma accuminata, and lymphadenopathy (Yang et al. 2013). In recent years, the species has been subject to a rapid demographic decline and ranked as ‘threatened’ on the China Species Red List (Wang and Xie 2004). It is thus urgent to take effective measures to conserve this Endangered endemic species. Herein, we first report and characterize its complete plastome based on Illumina paired-end sequencing data, which will contribute to the further studies on its genetic research and resource utilization. The annotated cp genome of *D. versipellis* has been deposited into GenBank with the accession number MN604379.

In this study, *D. versipellis* was sampled from in Guangxi Zhuang Autonomous Region of China, located at 108°49′59″E, 24°49′42″N. A voucher specimen (Y.-C. Shi et al. H346) was deposited in the Guangxi Key Laboratory of Plant Conservation and Restoration Ecology in Karst Terrain, Guangxi Institute of Botany, Guangxi Zhuang Autonomous Region and Chinese Academy of Sciences, Guilin, China. The experiment procedure is as reported in Zhang et al. (2019). Around 2 Gb clean data were used for the cp genome *de novo* assembly by the program NOVOPlasty (Dierckxsens et al. 2017) and direct-viewing in Geneious R11 (Biomatters Ltd., Auckland, New Zealand). Annotation was performed with the program Plann (Huang and Cronk 2015) and Sequin (http://www.ncbi.nlm.nih.gov/).

The chloroplast genome of *D. versipellis* is a typical quadripartite structure with a length of 156,735 bp, which contained inverted repeats (IR) of 25,925 bp separated by a large single-copy (LSC) and a small single-copy (SSC) of 86,514 bp and 18,371 bp, respectively. The cpDNA contains 133 genes, comprising 86 protein-coding genes, 37 tRNA genes, 8 rRNA genes, and 2 processed pseudogenes. Among the annotated genes, 16 of them contain one intron (*atp*F, *ndh*A, *ndh*B, *rps*16, *rpoC*1, *pet*B, *pet*D, *rpl*16, *rpl*2, *trn*A-UGC, *trn*I-GAU, *trn*H-GUG, *trn*G-UCC, *trn*K-UUU, *trn*L-UAA, and *trn*V-UAC), and 3 genes (*clp*P, *rps*12, and *ycf*3) contain two introns. The overall GC content of the plastome is 38.5%.

To identify the phylogenetic position of *D. versipellis*, phylogenetic analysis was conducted. A neighbor-joining (NJ) tree with 1000 bootstrap replicates was inferred using MEGA version 7 (Kumar et al. 2016) from alignments created by the MAFFT (Katoh and Standley 2013) using plastid genomes of 17 species. It showed the position of *D. versipellis* was close to the congeneric *D. pleiantha* (Figure 1). Our findings can be further used for plastome evolution and phylogenomic studies of Berberidaceae. It will also provide fundamental data for the utilization and management of this important medicinal plant.

**Figure 1.**
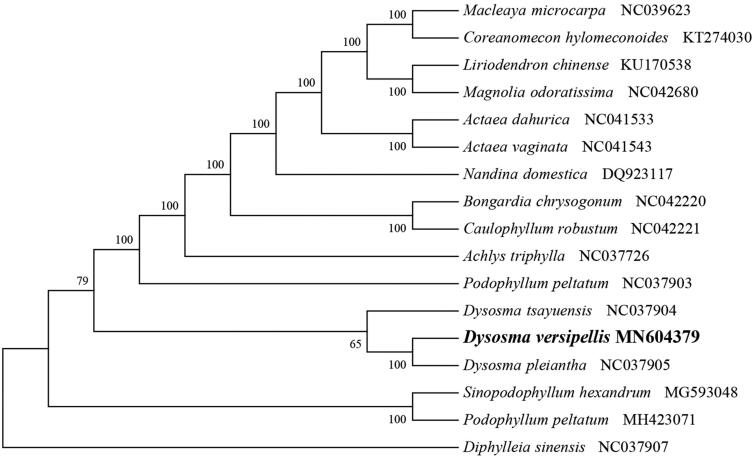
NJ phylogenetic tree of *D. versipellis* with 16 species was constructed by chloroplast plastome sequences. Numbers on the nodes are bootstrap values from 1000 replicates. *Diphylleia sinensis* was selected as outgroups.

## References

[CIT0001] Dierckxsens N, Mardulyn P, Smits G 2017 NOVOPlasty: *de novo* assembly of organelle genomes from whole genome data. Nucleic Acids Res. 45(4):e18. 28204566 10.1093/nar/gkw955 PMC5389512

[CIT0002] Huang DI, Cronk Q 2015 Plann: a command-line application for annotating plastome sequences. Appl Plant Sci. 3(8):1500026. 10.3732/apps.1500026 PMC4542940 26312193

[CIT0003] Katoh K, Standley DM 2013 MAFFT multiple sequence alignment software version 7: improvements in performance and usability. Mol Biol Evol. 30(4):772–780. 23329690 10.1093/molbev/mst010 PMC3603318

[CIT0004] Kumar S, Stecher G, Tamura K 2016 MEGA7: molecular evolutionary genetics analysis Version 7.0 for bigger datasets. Mol Biol Evol. 33(7):1870–1874. 27004904 10.1093/molbev/msw054 PMC8210823

[CIT0005] Wang S, Xie Y 2004 China species red list. Beijing: Higher Education Press.

[CIT0007] Yang Z, Liu XM, Wang KW, Cao XJ, Wu SH 2013 Novel linear and step-gradient counter-current chromatography for bio-guided isolation and purification of cytotoxic podophyllotoxins from *Dysosma versipellis* (Hance). J Sep Sci. 36(6):1022–1028. 23418155 10.1002/jssc.201201038

[CIT0008] Zhang D, Shao J, Li D 1991 A study on the karyotypes of *Dysosma versipellis* and *D. pleiantha* endemic to China. Guihaia. 11(1):58–62.

[CIT0009] Zhang Y, Shi YC, Duan N, Liu BB, Mi J 2019 Complete chloroplast genome of *Euphorbia tirucalli* (Euphorbiaceae), a potential biofuel plant. Mitochondrial DNA Part B. 4(1):1973–1974.

